# Metabarcoding study of potential pathogens and zoonotic risks associated with dog feces in Seoul, South Korea

**DOI:** 10.1371/journal.pntd.0012441

**Published:** 2024-08-28

**Authors:** Isuru Liyanagama, Singeun Oh, Jun Ho Choi, Myung-hee Yi, Myungjun Kim, Sohyeon Yun, Dongjun Kang, Soo Lim Kim, Maria Gloria Ojeda Ayala, Fred Odua, Tai-Soon Yong, Ju Yeong Kim

**Affiliations:** 1 Department of Global Health and Disease Control, Graduate School of Public Health, Yonsei University, Seoul, Republic of Korea; 2 Department of Animal Production and Health, Kandy, Sri Lanka; 3 Department of Tropical Medicine, Institute of Tropical Medicine, Arthropods of Medical Importance Resource Bank, Yonsei University College of Medicine, Seoul, Republic of Korea; 4 Department of Tropical Medicine, Graduate School of Medical Science, Brain Korea 21 Project, Yonsei University College of Medicine, Seodaemun-gu, Seoul, South Korea; 5 Production Department, Nakasongola, Uganda; University of Sao Paulo: Universidade de Sao Paulo, BRAZIL

## Abstract

**Background:**

A significant portion of South Korea’s population, approximately a quarter, owns pets, with dogs being the most popular choice among them. However, studies analyzing the fecal organism communities of dogs in South Korea are lacking, and limited efforts have been exerted to identify pathogens with potential zoonotic implications. Therefore, this study aimed to investigate potential pathogens using metabarcoding analysis and evaluate the risk of zoonotic diseases in dog feces in Seoul, South Korea.

**Methodology:**

Fecal samples were collected from both pet and stray dogs in the Mapo district of Seoul. Next-generation sequencing (NGS) was utilized, employing 16S rRNA amplicon sequencing to identify prokaryotic pathogens, and 18S rRNA amplicon sequencing for eukaryotic pathogens. The data obtained from the QIIME2 pipeline were subjected to various statistical analyses to identify different putative pathogens and their compositions.

**Principal findings:**

Significant variations in microbiota composition were found between stray and pet dogs, and putative prokaryotic and eukaryotic pathogens were identified. The most prevalent putative bacterial pathogens were *Fusobacterium*, *Helicobacter*, and *Campylobacter*. The most prevalent putative eukaryotic pathogens were *Giardia*, *Pentatrichomonas*, and *Cystoisospora*. Interestingly, *Campylobacter*, *Giardia*, and *Pentatrichomonas* were found to be significantly more prevalent in stray dogs than in pet dogs. The variation in the prevalence of potential pathogens in dog feces could be attributed to environmental factors, including dietary variances and interactions with wildlife, particularly in stray dogs. These factors likely contributed to the observed differences in pathogen occurrence between stray and pet dogs.

**Conclusions/Significance:**

This study offers valuable insights into the zoonotic risks associated with dog populations residing in diverse environments. By identifying and characterizing putative pathogens in dog feces, this research provides essential information on the impact of habitat on dog-associated pathogens, highlighting the importance of public health planning and zoonotic risk management.

## Author summary

The ownership of pets, especially dogs, is widespread in South Korea, with millions of people choosing them as companions. However, there is a lack of research on the composition of organisms from dog feces and the identification of potential disease-causing pathogens that can affect both dogs and humans. Understanding these risks is crucial as many infectious diseases can be transmitted from animals to humans. In this study, we used metabarcoding techniques to analyze the fecal organisms of pet and stray dogs in Seoul, South Korea. We found significant differences in the microbiota composition between stray and pet dogs, and we identified putative bacterial pathogens, such as *Helicobacter*, *Campylobacter*, and *Fusobacterium*, and eukaryotic pathogens, such as *Giardia*, *Pentatrichomonas*, and *Cystoisospora*. The prevalence of these putative pathogens was higher in stray dogs compared to pet dogs. These findings emphasize the importance of public health planning and zoonotic disease control, particularly in densely populated urban areas where the risk of zoonotic pathogens associated with dogs is amplified. It is crucial to educate pet owners and the general public about the potential risks associated with exposure to dog feces and contaminated environments.

## Introduction

According to the World Health Organization, zoonoses are diseases or infections that can be naturally transmitted from animals to humans and vice versa [1]. Domestic animals such as cattle, sheep, goats, dogs, cats, horses, and pigs can act as reservoirs of pathogens and transmit diseases to humans [2], and approximately 61% of human pathogens are zoonotic [3].

Human population density is a significant predictor of emerging infectious diseases, including those caused by non-wildlife zoonotic pathogens [4]. The increase in the population of stray and semi-domestic dogs in urban areas has also contributed to the risk of zoonotic diseases [5]. However, the role of companion animals, particularly dogs and cats, in public health and zoonotic disease spread is often overlooked [6]. In South Korea, many households own pets, with dogs being one of the most common companion animals [7]. Public parks can be a source of zoonotic infections, particularly when contaminated soil is ingested, and stray dogs roaming in public areas can facilitate the transmission of zoonotic pathogens [8,9].

Seoul, the capital city of South Korea, exhibits high population density [10], which renders it susceptible to the emergence of zoonotic diseases [4]. Several studies have explored the application of next-generation sequencing (NGS) in animal fecal metabarcoding [11–15]. However, to our knowledge, this study is the first attempt to metabarcode prokaryotic and eukaryotic communities concurrently in dogs, thereby enabling the investigation of their potential interactions. Furthermore, research on potential pathogens and their composition in dog feces from various living environments in Seoul remains scarce.

Therefore, the objective of this study was to utilize NGS to identify potential pathogens and evaluate the current risk of zoonosis in the feces of pet and stray dogs in Seoul. Specifically, we aimed to compare the microbial and parasitic communities between pet dogs and stray dogs to understand how different living conditions influence the prevalence of potential pathogens. The findings of this study provide valuable insights into the prevalence and potential hazards related to the environment of the dogs, thus enhancing our understanding of zoonotic risks in the city. To the best of our knowledge, this is the first study to employ such an approach on dogs in Seoul.

## Materials and methods

### Ethics statement

This study was conducted in strict accordance with the guidelines outlined by the Institutional Animal Care & Use Committee (IACUC) of South Korea, as specified by the Joint of Food and Drug Administration and Ministry of Agriculture, Food and Rural Affairs.

### Sample collection and DNA preparation from samples

A total of 41 dog fecal samples were collected between April and May 2022 from pet cafes (n = 16, pet group) and an animal shelter (n = 25, stray group) in Mapo-gu, Seoul, South Korea. The pet dogs were housed in three different pet cafes, where they were brought daily by their owners to spend leisure time with their pets, along with some pets owned by the cafe owners. Five samples were collected from one cafe, ten from another, and one from the third cafe. These cafes provide a relaxed environment for both pets and owners, allowing dogs to interact freely with other dogs and people during the day. The dogs in pet cafes typically return home with their owners at the end of the day, ensuring they have a stable and caring home environment. In contrast, the stray dogs were captured from the streets and wild areas around the city and brought to the animal shelter to give these animals a chance for a better life through rehabilitation and adoption into new homes. The shelter houses stray animals for a minimum of ten days and up to three months, during which efforts are made to find them new owners. While shelters provide basic care, including food, water, and medical attention, the level of individual attention and care is often less than that provided in pet cafes.

We closely observed the dogs until they excreted their feces to avoid cross-contamination and the risk of accidental cosampling. The samples were collected immediately after excretion. Stool samples were shipped to the laboratory within a day of collection, and DNA was extracted immediately using the FastDNA SPIN kit for soil (MP Biomedicals, Carlsbad, CA, USA), as referenced in previous studies, and then stored at -80°C until further use [16–19].

### Amplification of 16S rRNA/18S rRNA gene

For prokaryotic studies, the 16S rRNA gene V4 region was amplified using polymerase chain reaction (PCR) with primers listed in Table 1 [20]. For eukaryotic studies, the 18S rRNA gene V9 region was amplified using PCR with primers detailed in Table 1 [21]. An eight-cycle amplification step was performed to add the multiplexing indices and Illumina sequencing adapters. Mixed amplicons were pooled and sequenced on an Illumina iSeq 100 sequencing system using the Illumina iSeq 100 i1 Reagent v2 kit (San Diego, CA, USA) in accordance with the manufacturer’s instructions.

**Table 1:** Primer Sequences Used in This Study.

Target	Gene		Primer	Sequence
Prokaryotes metabarcoding	16S rRNA		Forward (515F)	5′-TCGTCGGCAGCGTCAGATGTGTATAAGA GACAGGTGCCAGCMGCCGCGGTAA-3′
			Reverse (806R)	5′-GTCTCGTGGGCTCGGAGATGTGTATAAG AGACAGGGACTACHVGGGTWTCTAAT-3′
Eukaryotes metabarcoding	18S rRNA		Forward (1391f)	5′-TCGTCGGCAGCGTCAGATGTGTATAAGAG ACAGGTACACACCGCCCGTC-3′
			Reverse (EukBr)	5′-GTCTCGTGGGCTCGGAGATGTGTATAAGA GACAGTGATCCTTCTGCAGGTTCACCTAC-3′
*Campylobacter jejuni*	*hipO*		Forward	5′-GAAGAGGGTTTGGGTGGTG-3′
			Reverse	5′-AGCTAGCTTCGCATAATAACTTG-3′
*Campylobacter coli*	*glyA*		Forward	5′-GTAAAACCAAAGCTTATCGTG-3′
			Reverse	5′-TCCAGCAATGTGTGCAATG-3′
*Campylobacter upsaliensis*	*glyA*		Forward	5′-AATTGAAACTCTTGCTATCC-3′
			Reverse	5′- TCATACATTTTACCCGAGCT-3′
*Pentatrichomonas* sp.	SSU rRNA	1^st^	Forward	5′-ATGGCGAGTGGTGGAATA-3′
			Reverse	5′-CCCAACTACGCTAAGGATT-3′
		2^nd^	Forward	5′-TGTAAACGATGCCGACAGAG-3′
			Reverse	5′-CAACACTGAAGCCAATGCGAGC-3′
*Giardia* sp.	*TPI*	1^st^	Forward	5′-AAATTATGCCTGCTCGTCG-3′
			Reverse	5′-CAAACCTTTTCCGCAAACC-3′
		2^nd^	Forward	5′-CCCTTCATCGGTGGTAACTT-3′
			Reverse	5′- GTGGCCACCACTCCCGTGCC-3′
	*β-giardin*	1^st^	Forward	5′-AAGCCCGACGACCTCACCCGC AGTGC-3′
			Reverse	5′- GAGGCCGCCCTGGATCTTCGAG ACGAC-3′
		2^nd^	Forward	5′-GAACGAACGAGATCGAGGTCCG-3′
			Reverse	5′-CTCGACGAGCTTCGTGTT-3′

### Bioinformatics and statistical analysis

For bioinformatics analysis, the standard DADA2 denoising pipeline [22] from Quantitative Insights Into Microbial Ecology (QIIME) 2 software version 2022.2 [23] was used for demultiplexing, forward and reverse paired-end read merges, quality filtering, and chimeric sequence removal to generate feature tables of amplicon sequence variants (ASVs). For the taxonomic classification of eukaryotic ASV sequences, all the sequences included in the NCBI nucleotide database (https://www.ncbi.nlm.nih.gov/nuccore/) were used to build a database of fungi and parasites [24]. To do this, we performed an advanced search for “18S rRNA” [25] and obtained sequences from the NCBI database. The reads were taxonomically classified using the classify-consensus-blast plugin within QIIME2. This process involved using a perc-identity parameter set at 0.95 for comparison against the SILVA 138 reference database [26], specifically for the classification of 16S rRNA. The NCBI database was utilized for the classification of 18S rRNA. The sequences of chordates and plants were removed. In addition, sequences with an ASV number of five or less were excluded as thresholds. Then, 16S rRNA-seq data were rarefied to a consistent depth of 10,000 sequences across all samples for further analysis. Alpha diversity, which refers to the richness and diversity of microbial communities within a habitat type (S1 Table), was quantified using richness (number of ASVs per sample) and the Shannon diversity index. A glossary of terms such as Alpha diversity and Beta diversity is provided in S1 Table to define key concepts used in this research [23,27–34]. The Wilcoxon rank-sum test was used to analyze differences in the number of observed species and the Shannon index between groups. For Beta diversity analysis, Bray–Curtis distance-based principal coordinate analysis (PCoA) and permutational multivariate analysis of variance (PERMANOVA) were performed. Analysis of microbiome composition (ANCOM) tests that account for sample variability were used to identify differentially abundant taxa in intergroup compositional data [28]. Linear discriminant analysis effect size (LEfSe) was used to identify the differential abundance of bacterial taxa in the fecal microbiota in relation to the presence or absence of specific parasites between the groups [35]. Statistical analysis was performed using the Yates-corrected chi-square test in R studio, version 2022 [36]. A p-value of < 0.05 was considered to indicate statistical significance. Box plots were generated to visualize the distribution of relative abundances for putative pathogen groups. For each sample, the microbial composition was normalized by calculating the relative abundance of each taxon. The “ggplot2” library in R was used to create the box plots [37].

### Species-specific PCR and Sanger sequencing

Species-specific PCR and Sanger sequencing were conducted on *Campylobacter*, *Pentatrichomonas*, and *Giardia* due to their critical role in understanding the prevalence and significance as zoonotic pathogens across different environments. PCR was utilized to identify the species of *Campylobacter*, *Pentatrichomonas*, and *Giardia*, employing species-specific primer pairs targeting each species’ specific gene. Detailed primer sequences are provided in Table 1 [38–42]. Subsequently, Sanger sequencing of the PCR amplicons was performed by Bionics Co. (Seoul, Korea), and the obtained sequences were compared to sequences deposited in GenBank using BLAST.

## Results

### Bacterial analysis of feces from pet and stray dogs

Amplicon deep sequencing targeting the bacterial 16S rRNA gene V4 region and the eukaryotic 18S rRNA gene V9 region was performed to analyze the fecal microbiota of pets (n = 16) and stray dogs (n = 25) in Seoul. In the prokaryotic analysis, the average number of reads assigned to each taxa was 49,177 and 178 taxa (ASV) (S2 Table). In the eukaryotic analysis, the average number of reads assigned to each taxon was 549 and 9 taxa (ASV) were detected (S3 Table). The ASV rarefaction curves of the bacterial 16S rRNA gene amplicon sequences are shown in S1 Fig. These observations, combined with the plateauing trend in the rarefaction curves for bacterial communities (S1 Fig) and the high average number of reads per taxon (49,177) in prokaryotic analyses (S2 Table), confirm that the sequencing depth achieved in this study was sufficient for conducting downstream alpha and beta diversity analyses.

**Fig 1:**
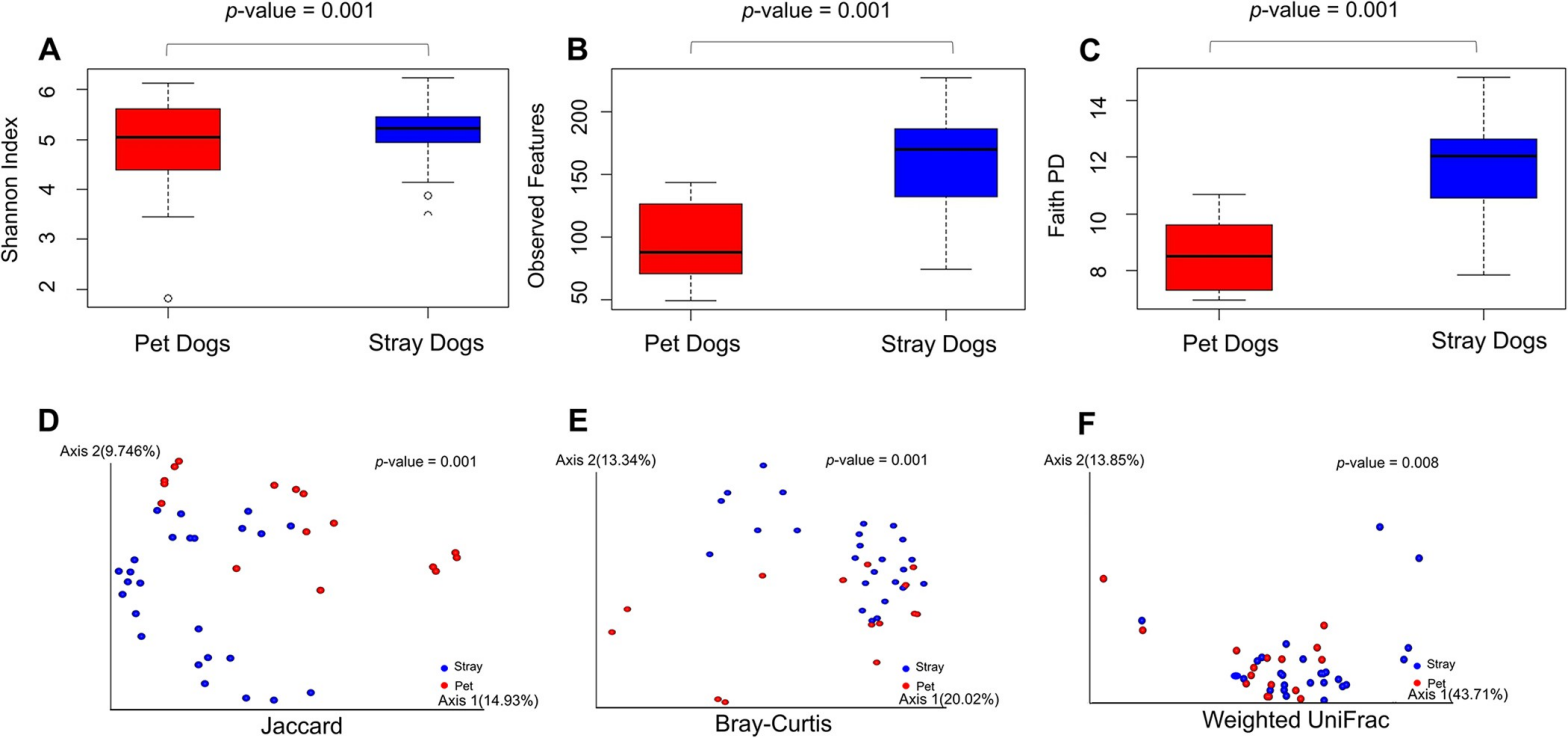
Visualization of alpha and beta diversity in the fecal microbiota of pet and stray dogs. (A) Shannon Index: Box plot comparing the Shannon index of microbial diversity between pet and stray dogs. The Shannon index measures alpha diversity, reflecting both the richness and evenness of species in a community. (B) Observed Features: Box plot comparing the number of observed features (Amplicon Sequence Variants, ASVs) between the two groups. This metric represents alpha diversity by counting the unique ASVs present in each sample, indicating species richness. (C) Faith’s Phylogenetic Diversity: Box plot comparing Faith’s phylogenetic diversity between pet and stray dogs. Faith’s phylogenetic diversity is an alpha diversity measure that considers the branch lengths of a phylogenetic tree, encompassing the evolutionary relationships between species. (D) Jaccard Index: PCoA plot illustrating the beta diversity based on the Jaccard index between pet and stray dogs. The Jaccard index measures beta diversity by comparing the presence or absence of species between samples, indicating community composition differences. (E) Bray–Curtis Index: PCoA plot showing the beta diversity based on the Bray–Curtis distance. The Bray–Curtis index assesses beta diversity by considering the abundance of species between samples, indicating community structure and dissimilarity. (F) Weighted UniFrac: PCoA plot representing the beta diversity based on the Weighted UniFrac distance. Weighted UniFrac is a beta diversity measure that accounts for both the presence/absence and the phylogenetic distances of species, indicating differences in community composition and evolutionary relationships. The statistical test conducted for alpha diversity indices (Shannon Index, Observed Features, Faith’s Phylogenetic Diversity) was the Wilcoxon rank-sum test. The statistical test conducted for beta diversity indices (Jaccard Index, Bray–Curtis Index, Weighted UniFrac) was PERMANOVA (Permutational Multivariate Analysis of Variance).

Alpha diversity was signified by three matrices: Shannon index, observed features, and Faith’s phylogenetic diversity. All alpha diversity indices were higher in the stray group than in the pet group (Fig 1A–1C). Wilcoxon rank-sum test results showed that the stray dogs had a significantly higher alpha diversity than the pet dogs (*p* = 0.001, 0.001, and 0.001, respectively). PCoA and PERMANOVA results on the beta diversity between the groups according to the Bray–Curtis distance, Jaccard, and Weighted UniFrac distances (Fig 1D–1F) showed that the microbial composition between the groups was significantly different (*p* = 0.001, 0.001, and 0.008, respectively).

**Fig 2:**
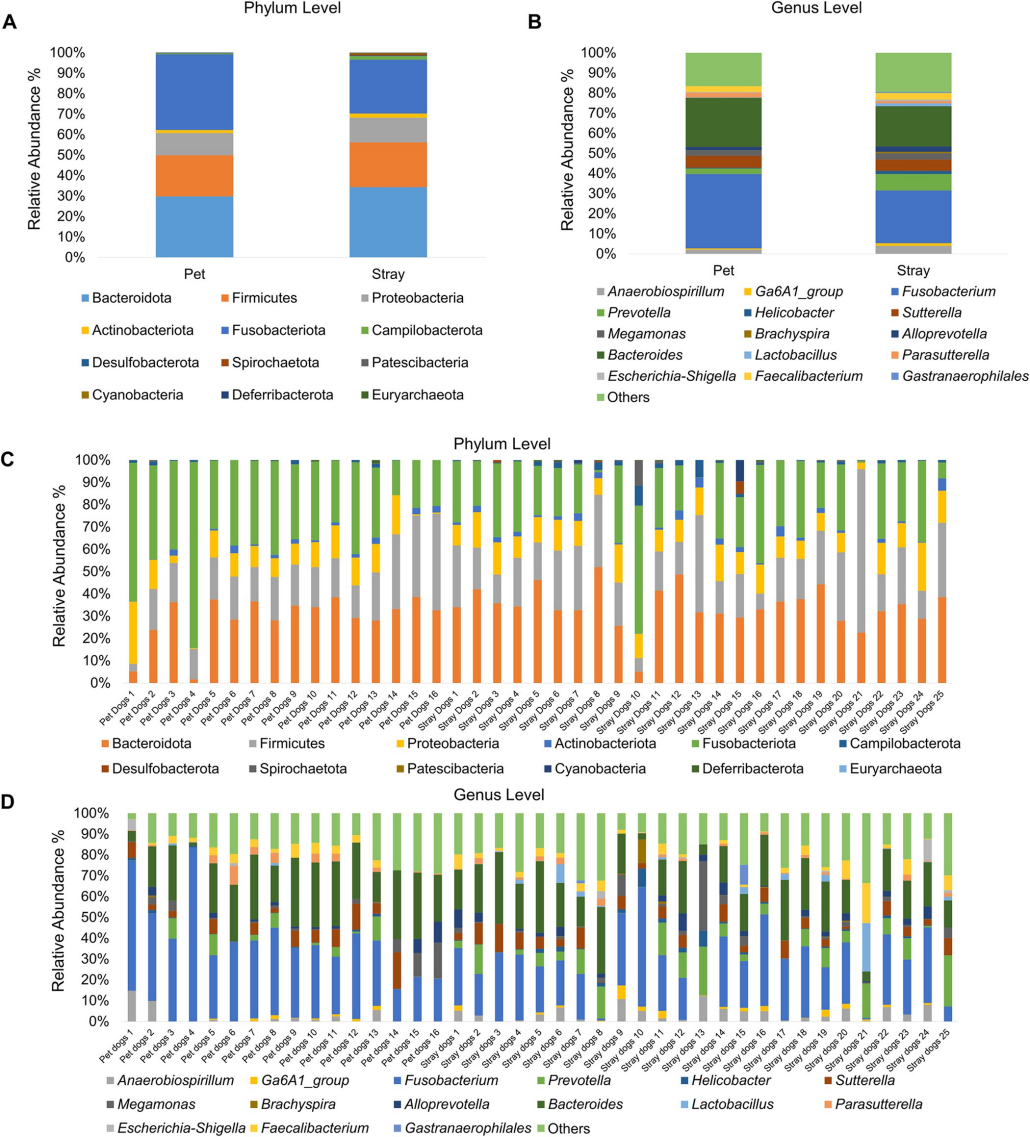
Average relative abundance of bacterial taxa in fecal samples. Bar plot showing the average relative abundance of bacterial phyla (A) and genera (B) in pet and stray dogs. Stacked bar plot illustrating the relative abundance of bacterial phyla (C) and genera (D) in individual fecal samples.

Taxonomic analysis results for 16S rRNA gene sequencing are shown in Fig 2. At the phylum level, Fusobacteriota (13.61%) was the most abundant in the pet group, followed by Bacteroidota (10.96%), and then Firmicutes (7.43%). By contrast, Bacteroidota (21.65%) was the most abundant in the stray group, followed by Fusobacteriota (16.00%), and then Firmicutes (13.83%) (Fig 2A and 2C). At the genus level, *Fusobacterium* was the most abundant in both groups (34.89% and 27.22% in the pet and stray groups, respectively), followed by *Bacteroides* (23.12% and 20.79% of in the pet and stray groups, respectively). *Prevotella* was the third most dominant genus in the stray group, representing 8.44% of the total sequences (Fig 2B and 2D).

**Fig 3:**
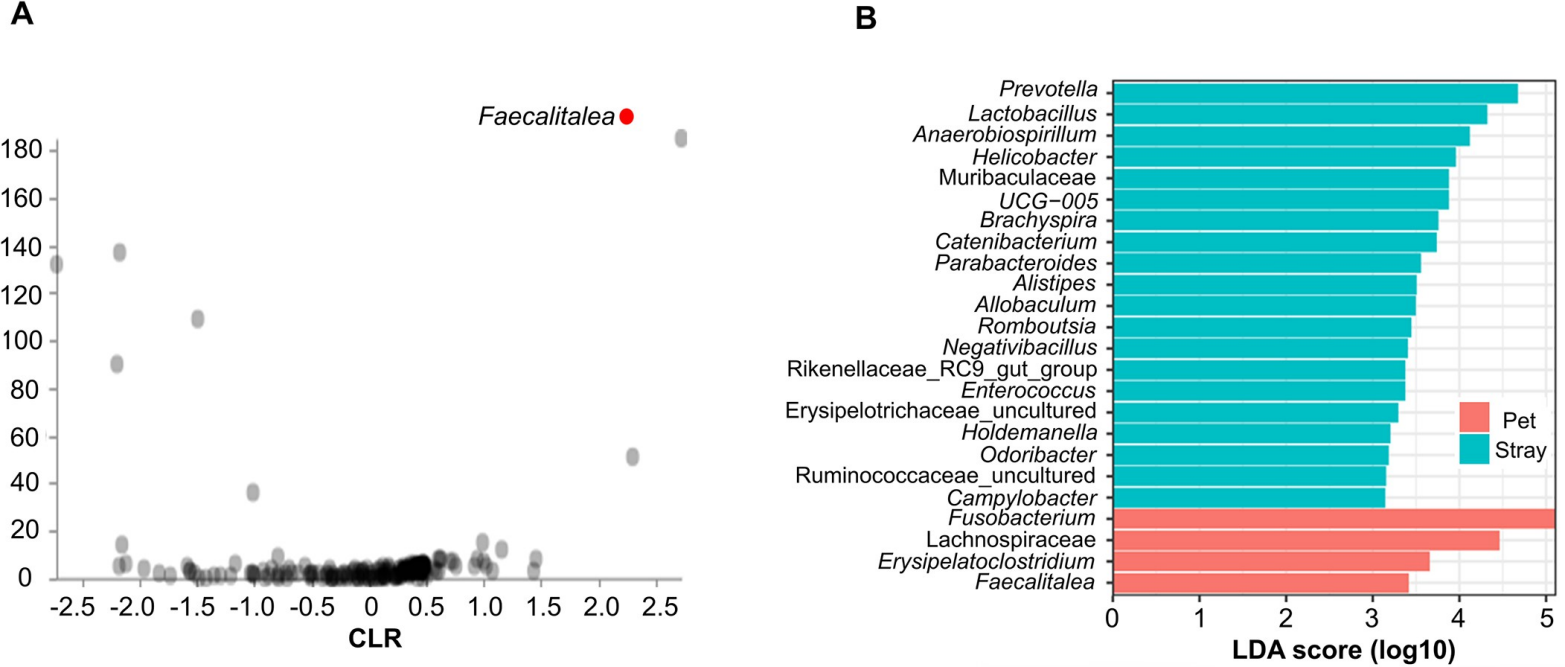
Differentially abundant microbial taxa between pet and stray dogs. (A) ANCOM Volcano Plot: Volcano plot illustrating differentially abundant taxa between pet and stray dogs. The red dot represents *Faecalitalea* with the highest W-statistic value of 185, indicating its significant differential abundance. (B) LEfSe Bar Plot: Bar plot showing taxa with LDA scores greater than 3, indicating significant differential abundance between pet and stray dogs.

The differentially abundant microbial taxa were identified using ANCOM. The ANCOM volcano plot illustrating the differential abundance between the pet and stray dog groups is shown in Fig 3A. In Fig 3A, the red dot indicates the highest W- statistic value of 185, which represented *Faecalitalea* as a significantly different taxon between the groups. The result illustrated that *Faecalitalea* was significantly more abundant in the pet group than in the stray group (S4 Table). LEfSe analysis also showed similar results. *Prevotella*, *Lactobacillus*, *Anaerobiospirillum*, and various other taxa were more abundant in the stray group than in the pet group, whereas *Fusobacterium*, *Lachnospiraceae*, *Erysipelatoclostridium and Faecalitalea* were more abundant in the pet group than in the stray group (Fig 3B).

The positivity from the taxonomic composition of the bacterial microbiota was analyzed, and 10 bacterial genera were identified as putative pathogens (Table 2). The prevalence was determined by considering the cumulative count of positive samples, surpassing the threshold of 5 reads for a specific genus. The difference in putative pathogen positivity between the two groups was analyzed using the chi-square test, and results showed that *Campylobacter* was significantly more prevalent in the stray group than in the pet group. The difference in abundance between the groups is shown in box plots (S2 Fig). To determine the specific risks associated with *Campylobacter*, species-specific PCR and Sanger sequencing were conducted. Among the 23 samples positive for *Campylobacter*, 14 were identified as *C*. *upsaliensis*, 3 as *C*. *coli*, and 1 as *C*. *jejuni* (one sample showed a mixed presence of *C*. *upsaliensis* and *C*. *coli*), all of which are associated with human infection (S5 Table).

**Table 2:** Positivity differences in putative bacterial pathogens between the stray and pet dog groups.

Genus	Pet (n = 16)	Stray (n = 25)	Total Prevalence (%)	p-value
*Fusobacterium*	16 (100%)	25 (100%)	100	1
*Streptococcus*	6 (37.5%)	6 (24.0%)	29.3	0.5653
*Campylobacter*	4 (25.0%)	19 (76.0%)	56.1	0.0039**
*Mycoplasma*	0 (0.0%)	5 (20.0%)	12.2	0.1557
*Enterococcus*	2 (12.5%)	11 (44.0%)	31.7	0.076
*Escherichia-Shigella*	9 (56.3%)	11 (44.0%)	48.8	0.6562
*Peptostreptococcus*	1 (6.3%)	3 (12.0%)	9.8	0.9475
*Helicobacter*	15 (93.8%)	21 (84.0%)	87.8	0.6589

*Test statistics: Yates-corrected chi-square test, statistically significant at a 95% confidence interval.

** *P* < 0.01, * *P* < 0.05.

### Eukaryotic analysis of feces from pet and stray dogs

The taxonomic analysis results for 18S rRNA gene sequencing are shown in S3 Table. It identified different categories of organisms: *Oesopagostomum* and *Trichuris* as helminthic parasites; *Tritrichomonas*, *Pentatrichomonas*, *Cystoisospora*, *Giardia*, and *Cryptosporidium* as protozoal parasites; and *Saccharomyces* and *Cyniclomyces* as fungi. The eukaryotic genera with the highest average relative abundance were *Tritrichomonas* and *Pentatrichomonas*, followed by *Giardia*, and then *Cyniclomyces* (S3 Fig). Most of the organisms identified through 18S rRNA sequencing were abundant in stray dogs. Analysis of 18S rRNA gene sequencing results revealed seven putative zoonotic parasites at the genus level (Table 3).

**Table 3:** Positivity differences in putative eukaryotic pathogens between the stray and pet groups based on 18S rRNA sequencing results.

Genus	Pet (n = 16)	Stray (n = 25)	Total Prevalence (%)	p-value
*Giardia*	2 (12.5%)	14 (56.0%)	39.0	0.014 *
*Cryptosporidium*	0 (0.0%)	1 (4.0%)	2.4	1.000
*Tritrichomonas*	1 (6.3%)	1 (4.0%)	4.9	1.000
*Pentatrichomonas*	0 (0.0%)	8 (32.0%)	19.5	0.034 *
*Cystoisospora*	0 (0.0%)	2 (8.0%)	4.9	0.677
*Oesophagostomum*	1 (6.3%)	0 (0.0%)	2.4	0.820
*Trichuris*	0 (0.0%)	1 (4.0%)	2.4	1.000

*Test statistics: Yates-corrected chi-square test, statistically significant at a 95% confidence interval.

** *P* < 0.01, * *P* < 0.05.

Among these putative pathogens, two were helminths, and the remaining five were protozoans. Further analyses were conducted to compare the putative pathogen positivity between the two groups. Yates-corrected chi-square test results showed that protozoan pathogens *Giardia* and *Pentatrichomonas* were significantly positive in the stray group when compared with the pet group. The difference in abundance between the groups is shown in box plots (S4 Fig). To determine the specific risks associated with them, *Pentatrichomonas* and *Giardia* species-specific PCR and Sanger sequencing were conducted. Among the 8 samples positive for *Pentatrichomonas*, 7 were identified as *P*. *hominis*, which is associated with human infection (S6 Table). Moreover, among the 16 samples positive for the *Giardia* genus, 14 were identified as *G*. *intestinalis*. Among the 14 samples positive for *G*. *intestinalis*, 8 strains belonged to assemblage D, 9 to assemblage C, and 2 to assemblage A. Four samples exhibited a mix of assemblages C and D, and 1 sample displayed a mix of assemblages A and C (S7 Table).

### Differential abundance analysis of microbiota in relation to eukaryotic pathogen presence

**Fig 4:**
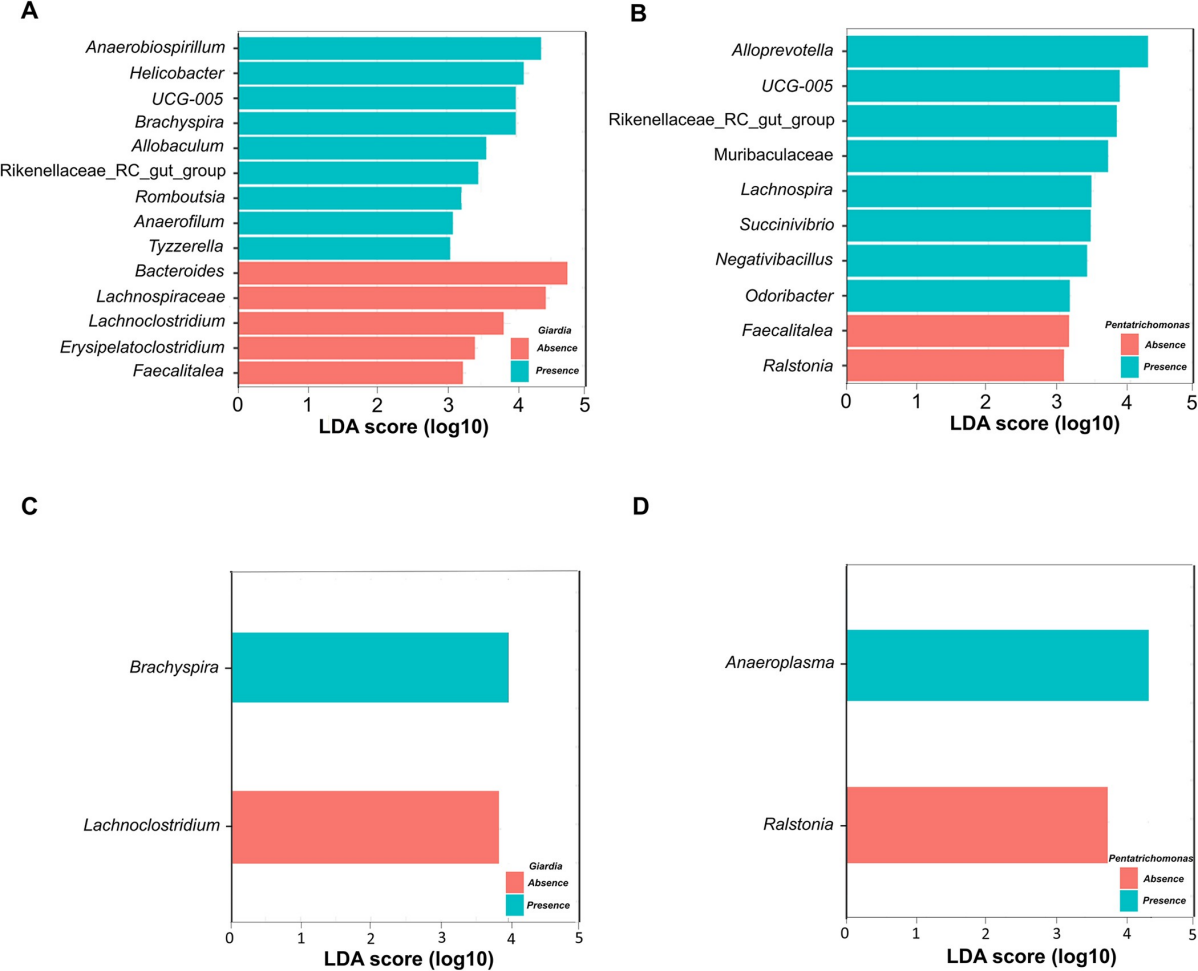
LEfSe analysis of differentially abundant taxa based on the presence or absence of *Giardia* and *Pentatrichomonas* in the feces. (A) Bar plot comparing the abundance of bacterial taxa between *Giardia*-present and *Giardia*-absent samples from both pet and stray dogs. (B) Bar plot comparing the abundance of bacterial taxa between *Pentatrichomonas*-present and *Pentatrichomonas*-absent samples from both pet and stray dogs. (C) Bar plot showing the differential abundance of bacterial taxa between *Giardia*-present and *Giardia*-absent samples within only stray dogs. Only genera with LDA scores more than 3 were selected. (D) Bar plot depicting the differential abundance of bacterial taxa between *Pentatrichomonas*-present and *Pentatrichomonas*-absent samples within only stray dogs. Only genera with LDA scores more than 3 were selected.

LEfSe analysis of the 16S rRNA sequencing data showed that *Anaerobiospirillum*, *Helicobacter*, *UCG-005*, *Allobaculum*, and *Romboutsia* were more abundant in the Giardia-present group than in the Giardia-absent group. In contrast, Bacteroides, *Lachnospiraceae*, *Lachnoclostridium*, *Erysipelatoclostridium*, and *Faecalitalea* were more abundant in the *Giardia*-absent group than in the *Giardia*-present group (Fig 4A). Meanwhile, *Alloprevotella*, *UCG-005*, *Muribaculaceae*, and *Odoribacter* were more abundant in the *Pentatrichomonas*-present group than in the *Pentatrichomonas*-absent group, whereas *Faecalitalea* and *Ralstonia* were more abundant in the *Pentatrichomonas*-absent group than in the *Pentatrichomonas*-present group (Fig 4B). *UCG-005* showed a positive association with the presence of both *Giardia* and *Pentatrichomonas*, whereas *Faecalitalea* had a negative association with these two protozoan organisms. Similarly, a separate LefSe analysis was performed considering the effect within the stray group alone to eliminate the effect of the environment. *Brachyspira* was more abundant in the *Giardia*-present group than in the *Giardia*-absent group, whereas *Lachnoclostridium* was more abundant in the *Giardia*-absent group than in the *Giardia*-present group (Fig 4C). Meanwhile, *Anaeroplasma* was more abundant in the *Pentatrichomonas*-present group than in the *Pentatrichomonas*-absent group, whereas *Ralstonia* was more abundant in the *Pentatrichomonas*-absent group than in the *Pentatrichomonas*-present group (Fig 4D).

## Discussion

In delineating the differences between the pet and stray groups, it is important to note that the pet group consisted of dogs in controlled home environments, receiving regular care, being fed by their owners, and having limited outdoor access. Specifically, these pet dogs were brought daily to pet cafes by their owners. In contrast, the stray group included dogs that initially lived in uncontrolled environments, such as streets or wild areas, before being captured and brought to an animal shelter. The shelter housed these dogs for varying periods, with the specifics of their prior uncontrolled environments remaining unmeasured due to limited information.

Notably, these distinctions were reflected in the significant differences found in microbiota composition between the stray and pet groups. Furthermore, our study identified putative prokaryotic and eukaryotic pathogens with varying prevalence rates between these two distinct groups. Yarlagadda et al. [43] investigated the microbiota of pet and stray dogs from South Africa and Laos and found that the alpha diversity of the pet dogs was significantly lower than that of the stray dogs from the two regions. This result is consistent with the present findings.

Previous studies have employed metabarcoding to explore the zoonotic risk of animals, including dog, revealing significant insights into the diversity and composition of microorganisms [44–49]. For instance, recent studies have utilized metabarcoding to detect zoonotic pathogens in controlled or uncontrolled environments, providing valuable data on the zoonotic risks [46–49]. These studies highlight the effectiveness of metabarcoding in uncovering complex microbial ecosystems and their potential zoonotic implications.

In the present study, 16S rRNA gene sequencing of 41 fecal samples indicated 12 bacterial taxa at the phylum level and 176 bacterial taxa at the genus level. These findings are consistent with those obtained by Hand et al. [50] and Deng P et al. [51]. Phyla Bacteroidetes, Fusobacteria, and Firmicutes and genera *Fusobacterium*, *Bacteroides*, *Prevotella*, and *Anaerobiospirillum* were identified in the fecal samples. Among these taxa, several putative pathogens that could have zoonotic implications for humans have been identified, thus presenting a significant public health risk. The most prevalent putative pathogen identified in our study was *Fusobacterium*, which was detected in 41 of 41 samples (100% positivity). Various species of *Fusobacterium* can cause a wide range of human diseases, including pericarditis, osteomyelitis, and periodontal diseases [52–54]. *Helicobacter* was the second most abundant putative bacterial pathogen, with a positivity rate of 36 samples (87.8%). Shaaban et al. (2023) detected *Helicobacter pylori* antigen in dog and human stool samples, with a prevalence of 78.4% [55]. *Helicobacter canis*, which can be transmitted to humans through dogs, has been associated with gastroenteritis in children [56]. These results suggest that dog feces can act as a potential source of infection for *Helicobacter* spp. in humans [56–58]. *Campylobacter*, the third most abundant putative bacterial pathogen, was detected in 23 (56%) samples. Interestingly, it was significantly more prevalent in the stray dog samples (76% positivity) than in the pet dog samples (25% positivity). This difference may be attributed to environmental conditions and dietary differences among the dogs, which will be discussed further. A previous study in Portugal identified dog ownership, particularly of puppies, as a risk factor for campylobacteriosis [59]. Other studies conducted across Europe have attributed 9%–25% of *Campylobacter* infections to pets [60–62]. Furthermore, *Escherichia_Shigella*, the fourth most prevalent putative pathogen, had a prevalence of 48.8% (20 of the 41 samples). Fecal contamination associated with unleashed or stray dogs in the USA has led to outbreaks of Shiga toxin-producing *Escherichia coli* in humans [63]. Additionally, *Enterococcus* was found in 13 fecal samples (31.7%). Certain species of *Enterococcus*, such as *E*. *faecium*, *E*. *faecalis*, and *E*. *hirae*, can colonize the human intestinal tract and contribute to antimicrobial resistance [64]. Of the 23 *Campylobacter*-positive samples, Sanger sequencing results revealed the presence of 3 *C*. *coli* isolates, 1 *C*. *jejuni* isolates, and 14 *C*. *upsaliensis* isolates. *C*. *coli* and *C*. *jejuni* are recognized as the most common causes of human campylobacteriosis, with symptoms ranging from mild diarrhea to Guillain–Barré syndrome [65–67]. While *C*. *coli* and *C*. *jejuni* are widely recognized as the most commonly encountered *Campylobacter* species in animals [66,68], *C*. *upsaliensis* was found as the predominant species in our study, consistent with findings of another prior investigation [69]. *C*. *upsaliensis* is an emerging *Campylobacter* species that has been associated with human gastroenteritis, particularly in young children [70,71].

In this study, 9 genera of eukaryotic organisms were identified using 18S rRNA amplicon sequencing. Among the 9 genera, five were of protozoa (*Giardia*, *Cryptosporidium*, *Trichomonas*, *Cystoisospora* and *Pentatrichomonas*), two of helminths (*Trichuris*, *Oesophagostomum*), and two of fungi (*Cyniclomyces*, *Saccharomyces*). The parasites were included as putative zoonotic pathogens in this study. *Giardia* was detected in 16 of the 41 samples (39.02%) in our study, with 2 samples from pet dogs and 14 samples from stray dogs. It was the most prevalent putative eukaryotic pathogen identified in this study. *Giardia* is reportedly the most common parasitic pathogen found in dogs and cats, followed by significant infections from ascarids and taeniids [72]. Raza et al. [73] found a higher prevalence of *Giardia* in stray dogs than in pet dogs, which is consistent with our findings. *Pentatrichomonas* was detected in eight samples (32%) from the stray group but was not found in the pet group. In the present study, *Cystoisospora* was found in two samples (8%) from stray dogs. A previous research has also reported a higher prevalence of *Cystoisospora* in stray dogs than in pet dogs, which aligns with our findings [74]. Some species, such as *Cystoisospora belli*, are opportunistic protozoa that can cause cystoisosporiasis in humans, with symptoms such as diarrhea, steatorrhea, abdominal pain, fever, and weight loss [75]. *Trichuris* and *Oesophagostomum* are helminthic eukaryotes with zoonotic potential. *Trichuris* is a well-known parasite that can infect humans through transmission from dogs [76–80]. *Oesophagostomum* primarily affects livestock such as goats, pigs, and cattle [80,81]. However, several species of *Oesophagostomum* are zoonotic pathogens in mammals, including humans and dogs [80–84]. Therefore, dogs pose a significant zoonotic risk as they can transmit helminths to humans through close association with household members and heavy contamination of the environment, including soil and waterways, with parasitic eggs and oocysts [85–87]. Some of these pathogens, such as *Giardia* cysts and *Cystoisospora* oocysts, are highly resistant to environmental conditions and can survive for extended periods, contributing to their persistence and increasing the potential risk for environmental transmission [88,89].

Among the 16 samples positive for *Giardia*, identified as *G*. *intestinalis* through Sanger sequencing, a diverse array of strain assemblages was revealed. Notably, 8 strains belonged to assemblage D, 9 to assemblage C, and 2 to assemblage A. Notably, 4 samples exhibited a mix of assemblages C and D, and 1 sample displayed a unique mix of assemblages A and C. This finding aligns with previous studies that have reported a similar distribution of *Giardia* assemblages in dogs [90,91], where assemblages C and D are predominantly found. *G*. *intestinalis*, a commonly known species of *Giardia*, is an important cause of gastrointestinal illness worldwide, manifesting symptoms such as diarrhea, abdominal discomfort, and malabsorption [91]. Moreover, specific assemblages of *G*. *intestinalis* have been associated with zoonotic potential, signifying their ability to transmit between humans and animals [92,93]. Assemblage A and, to a lesser extent, assemblage B have been reported in human infections, often linked with waterborne outbreaks and close contact with animals [92,93]. Such findings underscore the importance of exploring the complex dynamics of *G*. *intestinalis* assemblages, not only in understanding human infections but also in elucidating their potential zoonotic implications and public health significance.

Out of the eight samples that yielded positive results for *Pentatrichomonas*, Sanger sequencing revealed the presence of seven *P*. *hominis* isolates. *P*. *hominis*, while generally considered a commensal protozoan in humans, has been implicated in rarely causing various adverse health effects, including diarrhea, pulmonary infections, and even rheumatoid arthritis [94–96]. Additionally, research suggests a correlation between *P*. *hominis* infection and colorectal cancer through microbiome alterations [97,98]. Therefore, the potential for zoonotic transmission of *P*. *hominis* should not be overlooked.

The significantly different prevalence between the groups (*Campylobacter*, *Giardia*, and *Pentatrichomonas*) can be attributed to several reasons. First is the exposure to contaminated environments. Stray dogs often have limited access to clean and controlled environments compared to pet dogs, which may result in contact with contaminated water sources, garbage, or other potential sources of pathogens [99–101]. This increases their likelihood of acquiring and shedding pathogens in their feces. Second is dietary differences between stray and pet dogs. Stray dogs may scavenge for food and consume contaminated or raw meat, carcasses, or other potential sources of pathogens [100–103]. This dietary behavior could elevate their exposure to the pathogen, consequently leading to a higher prevalence in their feces. Last is the interactions of stray dogs with wildlife. Stray dogs may have more frequent contact with wildlife, such as birds, rodents, or other animals, which can carry potential pathogens [104–107]. These interactions may provide opportunities for the transmission and colonization of bacteria in the gastrointestinal tract of dogs, ultimately resulting in a higher shedding rate in their feces.

Interestingly, some parasites require alterations in the host microbiota to promote their successful survival and control their numbers [108,109]. The host microbiota functions as a resistance factor against parasitic infection [110]. Therefore, we compared the microbial composition and pathogen prevalence between pet and stray dogs. Using ANCOM, we found significantly different bacterial taxa between the groups. *Faecalitalea* belonging to the phylum Firmicutes can ferment d-glucose, sucrose, d-mannose, and raffinose; the main end product of metabolism is butyric acid, which promotes postprandial insulin secretion and improves insulin response in patients with diabetes [111]. Fiber-rich ingredients promote the growth of beneficial bacteria, including *Faecalitalea* [112]. In line with this, we could assume that dog food which would have higher amount of fiber would affect the *Faecalitalea* abundance considering the pet group could have more well balanced dietfood as they have their owners who provide them with sincere care [113–117].

LEfSe analysis based on the presence of *Giardia* and *Pentatrichomonas*, which were significantly more prevalent in the stray group than in the pet group, revealed differentially abundant taxa. To obtain more precise insights into the effects of parasites on the host microbiota, we conducted separate analyses excluding data from the pet group. In the *Giardia*-present group, the only taxon remaining after accounting for habitat conditions was *Brachyspira*, a genus of multiple bacterial species that can cause diseases in various animals, including pigs, chickens, and humans [118]. Although *Brachyspira* has the potential to be a zoonotic pathogen, further investigation is required to examine *Brachyspira* specifically in relation to dog feces and explore potential correlations with *Giardia*.

This study has some limitations. Parasitic worms or eggs were not collected and identified under a microscope, and the Illumina iSeq 100 system can only identify species at the genus level. While NGS methods offer advantages in terms of cost-effectiveness and faster turnaround times for large studies, may have limitations in accuracy for certain pathogen species compared to some classical methods [119–123]. Future studies that combine classical methods, such as microscopic investigation, will be necessary to validate the findings of the current study. Additionally, the integration of shotgun metagenomics could offer more comprehensive and less biased data for bacterial and parasite communities at a higher resolution (species or strain level), which is crucial for assessing zoonotic risks [124–128].

Moreover, the challenges in acquiring specific breed data due to the predominant presence of mixed-breed stray dogs and the unavailability of comprehensive environmental records at the shelter limited our ability to conduct detailed breed-based or environmental diversity analyses. Additionally, factors such as the captured time point and sheltering period of the stray group, which might influence dietary behavior changes in sheltered environments, were not controlled in this study. Given these limitations, while this study suggests a potential relationship between pathogens and the environment, direct demonstration of this connection was not attained. Therefore, further research is needed to address these limitations and strengthen the findings of this study.

Despite its limitations, this study is the first to screen putative pathogens and analyze their prevalence based on habitat status by using metabarcoding. This study showed a possible link between changes in environmental conditions and putative pathogen prevalence. Overall, this study provides essential information regarding the potential effects of habitat on dogs and the risks associated with them. This research lays the groundwork for strategic public health planning and controlling zoonotic diseases in dogs.

## Supporting information

S1 Fig Rarefaction curves of 16S rRNA sequencing results using three diversities indices. (A) Observed Features Index: Rarefaction curve showing the number of observed features (ASVs) at different sequencing depths. (B) Shannon Index: Rarefaction curve illustrating the Shannon index at different sequencing depths. (C) Faith’s Phylogenetic Diversity: Rarefaction curve depicting Faith’s phylogenetic diversity at different sequencing depths.
(TIF)

S2 Fig Box plots displaying the relative abundance of putative pathogens identified from 16S rRNA results in both the pet and stray dog groups. (A) *Fusobacterium*: Box plot showing the relative abundance of *Fusobacterium*. (B) *Streptococcus*: Box plot showing the relative abundance of *Streptococcus*. (C) *Campylobacter*: Box plot showing the relative abundance of *Campylobacter*. (D) *Mycoplasma*: Box plot showing the relative abundance of *Mycoplasma*. (E) *Enterococcus*: Box plot showing the relative abundance of *Enterococcus*. (F) *Escherichia-Shigella*: Box plot showing the relative abundance of *Escherichia-Shigella*. (G) *Peptostreptococcus*: Box plot showing the relative abundance of *Peptostreptococcus*. (H) *Helicobacter*: Box plot showing the relative abundance of *Helicobacter*.
(TIF)

S3 Fig Average relative sequence abundance of eukaryotic taxa in fecal samples. (A) Group Comparison: Bar chart showing the average relative abundance of eukaryotic taxa (fungi, protozoa, and helminths) in the pet group, stray group, and both groups combined. (B) Individual Samples: Stacked bar plot illustrating the relative abundance of eukaryotic taxa in individual fecal samples.
(TIF)

S4 Fig Box plots displaying the relative abundance of putative pathogens identified from 18S rRNA results in both the pet and stray dog groups. (A) *Giardia*: Box plot showing the relative abundance of *Giardia*. (B) *Cryptosporidium*: Box plot showing the relative abundance of *Cryptosporidium*. (C) *Tritrichomonas*: Box plot showing the relative abundance of *Tritrichomonas*. (D) *Pentatrichomonas*: Box plot showing the relative abundance of *Pentatrichomonas*. (E) *Cystoisospora*: Box plot showing the relative abundance of *Cystoisospora*. (F) *Oesophagostomum*: Box plot showing the relative abundance of *Oesophagostomum*. (G) *Trichuris*: Box plot showing the relative abundance of *Trichuris*.
(TIF)

S1 Table Glossary of terms used in this research and their explanations. (DOCX)

S2 Table List of taxa of fecal microbiota in dogs. (XLSX)

S3 Table List of taxa of fecal eukaryota in dogs. (XLSX)

S4 Table ANCOM statistical results. (XLSX)

S5 Table Comparison of the target gene sequence of *Campylobacter* between the Sanger sequencing result and the best match obtained from NCBI blastn analysis. (DOCX)

S6 Table Comparison of the target gene sequence of *Pentatrichomonas* between the Sanger sequencing result and the best match obtained from NCBI blastn analysis. (DOCX)

S7 Table Comparison of the target gene sequence of *Giardia* between the Sanger sequencing result and the best match obtained from NCBI blastn analysis. (DOCX)
