# Supplementary figures and images for: Metabarcoding study of potential pathogens and zoonotic risks associated with dog feces in Seoul, South Korea
Source: PLoS Negl Trop Dis. 2024 Aug 28;18(8):e0012441. doi: 10.1371/journal.pntd.0012441 (PMC11355564; doi:10.1371/journal.pntd.0012441)

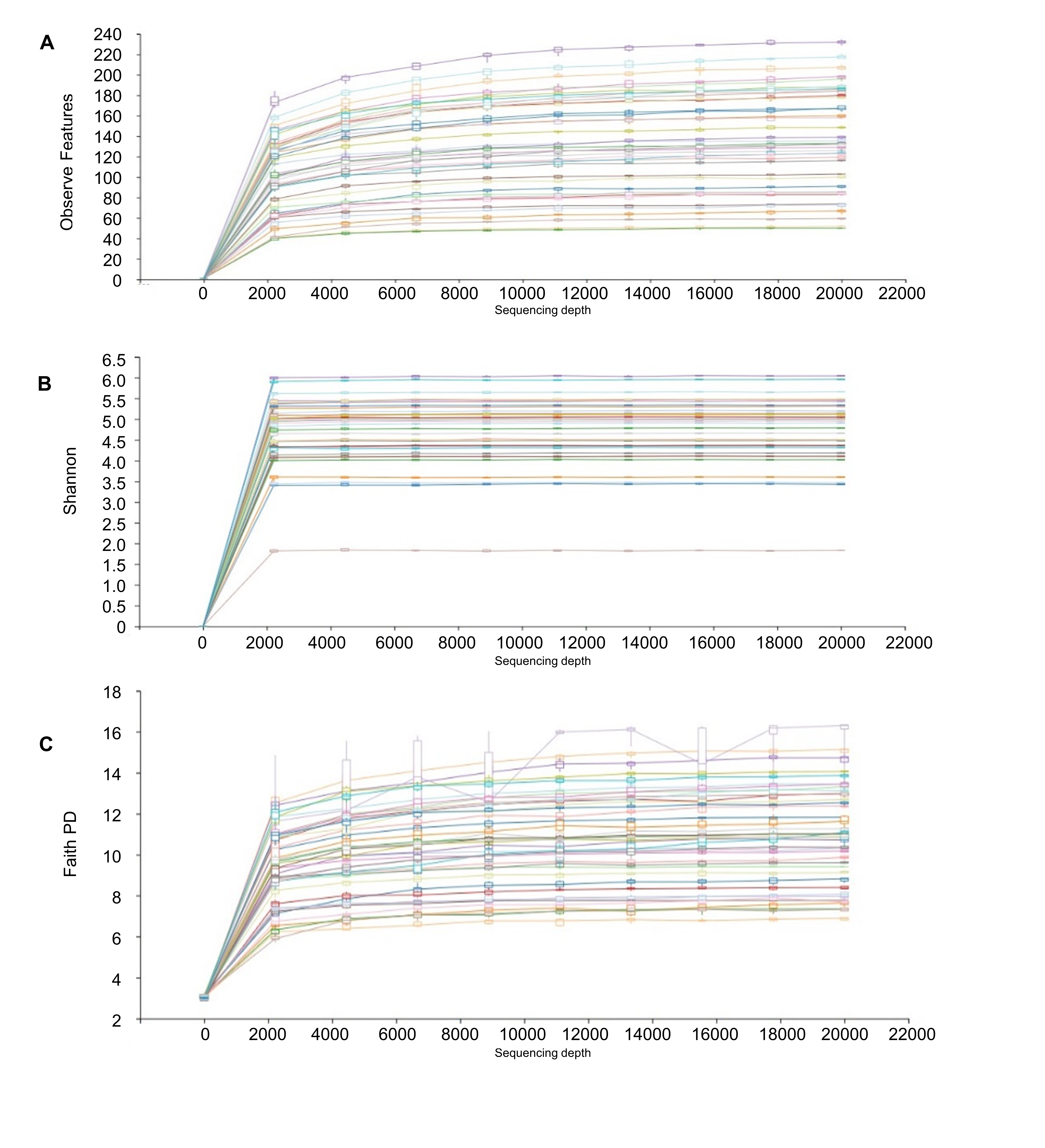

Supplement: S1 Fig — (A) Observed Features Index: Rarefaction curve showing the number of observed features (ASVs) at different sequencing depths. (B) Shannon Index: Rarefaction curve illustrating the Shannon index at different sequencing depths. (C) Faith’s Phylogenetic Diversity: Rarefaction curve depicting Faith’s phylogenetic diversity at different sequencing depths. (TIF) [file pntd.0012441.s001.TIF]
