# Supplementary material for: Metabarcoding study of potential pathogens and zoonotic risks associated with dog feces in Seoul, South Korea
Source: PLoS Negl Trop Dis. 2024 Aug 28;18(8):e0012441. doi: 10.1371/journal.pntd.0012441 (PMC11355564; doi:10.1371/journal.pntd.0012441)
Supplement: S1 Table — (DOCX) [file pntd.0012441.s005.docx]

Supplementary table 1. Glossary of terms used in this research and their explanations

| Glossary | Explanation | Reference |
| --- | --- | --- |
| Alpha diversity | The measure of observed richness (number of taxa) or evenness (the relative abundances of those taxa) of an average sample within a habitat type. | [27] |
| Beta diversity | The variability in community composition (the identity of taxa observed) among samples within a habitat | [27] |
| ANCOM  (Analysis of Composition of Microbiomes) | A statistical framework designed to account for the underlying structure in microbiome data. ANCOM is used for comparing the composition of microbiomes across two or more populations | [28] |
| ASVs  (Amplicon Sequence Variants) | Clusters of identical rRNA gene sequences, representing a precise and refined operational definition of a taxonomic unit in microbial community analysis. | [29] |
| PCoA  (Principal Coordinates Analysis) | PCoA (Principal Coordinates Analysis): A form of classic or metric multidimensional scaling that enables researchers to visualize variation across samples. By projecting observations into a lower-dimensional space, PCoA helps identify clusters and patterns within the data. | [30] |
| PERMANOVA  (Permutational Multivariate Analysis of Variance) | PERMANOVA (Permutational Multivariate Analysis of Variance based on distances): A statistical method that aims to identify covariates that could significantly explain the inter-subject variability captured by the pairwise distances. | [31] |
| QIIME 2  (Quantitative Insights Into Microbial Ecology version 2) | QIIME2 (Quantitative Insights into Microbial Ecology Version 2) as a widely used software platform for analyzing rRNA data, which is a common method for studying the composition of microbiomes. | [32] |
| e-value | E-value: A convenient measure of the statistical significance of database hits. In the context of sequence similarity detection by massive screening of local alignments to database sequences, the E-value of an alignment score can be interpreted as the expected number of false positives having this score or a higher one. potential impact of unobserved variables | [33] |
| LDA  (Linear Discriminant Analysis) | Linear Discriminant Analysis (LDA): A method that easily handles cases with unequal within-class frequencies and has been evaluated using randomly generated test data. LDA maximizes the ratio of between-class variance to within-class variance in any particular data set, thereby ensuring maximal separability. | [34] |
| LEfSe  (Linear Discriminant Analysis Effect Size) | LEfSe (Linear Discriminant Analysis Effect Size): A statistical method that determines the features (such as organisms, clades, operational taxonomic units, genes, or functions) most likely to explain differences between classes. LEfSe couples standard tests for statistical significance with additional tests that encode biological consistency and effect relevance, providing a comprehensive approach to identifying important features. | [35] |
