# Supplementary material for: Metabarcoding study of potential pathogens and zoonotic risks associated with dog feces in Seoul, South Korea
Source: PLoS Negl Trop Dis. 2024 Aug 28;18(8):e0012441. doi: 10.1371/journal.pntd.0012441 (PMC11355564; doi:10.1371/journal.pntd.0012441)
Supplement: S5 Table — (DOCX) [file pntd.0012441.s009.docx]

Table S5. Comparison of the target gene sequence of *Campylobacter* between the Sanger sequencing result and the best match obtained from NCBI blastn analysis.

| Sample  No. | Identified Species | Primers | Percent Identity | Best Matches in GenBank |
| --- | --- | --- | --- | --- |
| Pet 9 | *Campylobacter upsaliensis* | F:AATTGAAACTCTTGCTATCC  R:TCATACATTTTACCCGAGCT | 99 | AF550643.1 |
| Pet 13 | *-* | - | - | Unidentified |
| Pet 15 | *C. upsaliensis* | F:AATTGAAACTCTTGCTATCC  R:TCATACATTTTACCCGAGCT | 99 | OU701459.1 |
| Pet 16 | - | - | - | Unidentified |
| Stray 2 | *C. jejuni* | F:GAAGAGGGTTTGGGTGGTG  R:AGCTAGCTTCGCATAATAACTTG | 99 | CP047482.1 |
| Stray 3 | *C. upsaliensis* | F:AATTGAAACTCTTGCTATCC  R:TCATACATTTTACCCGAGCT | 99 | KU855045.1 |
| Stray 4 | *C. upsaliensis* | F:AATTGAAACTCTTGCTATCC  R:TCATACATTTTACCCGAGCT | 99 | CP053849.1 |
| Stray 5 | *C. upsaliensis* | F:AATTGAAACTCTTGCTATCC  R:TCATACATTTTACCCGAGCT | 95 | LR134372.1 |
| Stray 6 | *C. upsaliensis* | F:AATTGAAACTCTTGCTATCC  R:TCATACATTTTACCCGAGCT | 99 | OU701459.1 |
| Stray 8 | - | - | - | Unidentified |
| Stray 9 | *C. upsaliensis* | F:AATTGAAACTCTTGCTATCC  R:TCATACATTTTACCCGAGCT | 99 | CP053849.1 |
| Stray 10 | - | - | - | Unidentified |
| Stray 11 | *C. upsaliensis* | F:AATTGAAACTCTTGCTATCC  R:TCATACATTTTACCCGAGCT | 99 | AF550645.1 |
| Stray 12 | *C. upsaliensis* | F:AATTGAAACTCTTGCTATCC  R:TCATACATTTTACCCGAGCT | 99 | CP053849.1 |
| Stray 14 | *C. upsaliensis* | F:AATTGAAACTCTTGCTATCC  R:TCATACATTTTACCCGAGCT | 99 | KU855045.1 |
| Stray 15 | *C. upsaliensis* | F:AATTGAAACTCTTGCTATCC  R:TCATACATTTTACCCGAGCT GTGTT | 99 | OU701459.1 |
|  | *C. coli* | F:GTAAAACCAAAGCTTATCGTG  R:TCCAGCAATGTGTGCAATG | 96 | CP038868.1 |
| Stray 16 | *C. coli* | F:GTAAAACCAAAGCTTATCGTG  R:TCCAGCAATGTGTGCAATG | 96 | CP038868.1 |
| Stray 17 | *C. coli* | F:GTAAAACCAAAGCTTATCGTG  R:TCCAGCAATGTGTGCAATG | 94 | CP038868.1 |
| Stray 19 | *-* | *-* | - | Unidentified |
| Stray 20 | *C. upsaliensis* | F:AATTGAAACTCTTGCTATCC  R:TCATACATTTTACCCGAGCT | 98 | CP053849.1 |
| Stray 21 | *-* | *-* | - | Unidentified |
| Stray 22 | *C. upsaliensis* | F:AATTGAAACTCTTGCTATCC  R:TCATACATTTTACCCGAGCT | 99 | OU701459.1 |
| Stray 23 | *C. upsaliensis* | F:AATTGAAACTCTTGCTATCC  R:TCATACATTTTACCCGAGCT | 99 | OU701459.1 |
