# Supplementary material for: Metabarcoding study of potential pathogens and zoonotic risks associated with dog feces in Seoul, South Korea
Source: PLoS Negl Trop Dis. 2024 Aug 28;18(8):e0012441. doi: 10.1371/journal.pntd.0012441 (PMC11355564; doi:10.1371/journal.pntd.0012441)
Supplement: S6 Table — (DOCX) [file pntd.0012441.s010.docx]

Table S6. Comparison of the target gene sequence of *Pentatrichomonas* between the Sanger sequencing result and the best match obtained from NCBI blastn analysis.

| Sample  No. | Identified Species | Primers | Percent Identity | Best Matches in GenBank |
| --- | --- | --- | --- | --- |
| Stray 5 | *Pentatrichomonas hominis* | *** Primary**  F:ATGGCGAGTGGTGGAATA  R:CCCAACTACGCTAAGGATT  ***Secondary**  F:TGTAAACGATGCCGACAGAG  R:CAACACTGAAGCCAATGCGAGC | 99 | MK881031.1 |
| Stray 11 | *P. hominis* | *** Primary**  F:ATGGCGAGTGGTGGAATA  R:CCCAACTACGCTAAGGATT  ***Secondary**  F:TGTAAACGATGCCGACAGAG  R:CAACACTGAAGCCAATGCGAGC | 99 | MK881031.1 |
| Stray 12 | *P. hominis* | *** Primary**  F:ATGGCGAGTGGTGGAATA  R:CCCAACTACGCTAAGGATT  ***Secondary**  F:TGTAAACGATGCCGACAGAG  R:CAACACTGAAGCCAATGCGAGC | 99 | MK881031.1 |
| Stray 14 | *P. hominis* | *** Primary**  F:ATGGCGAGTGGTGGAATA  R:CCCAACTACGCTAAGGATT  ***Secondary**  F:TGTAAACGATGCCGACAGAG  R:CAACACTGAAGCCAATGCGAGC | 99 | KC594038.1 |
| Stray 16 | *P. hominis* | *** Primary**  F:ATGGCGAGTGGTGGAATA  R:CCCAACTACGCTAAGGATT  ***Secondary**  F:TGTAAACGATGCCGACAGAG  R:CAACACTGAAGCCAATGCGAGC | 99 | MH997492.1 |
| Stray 19 | *P. hominis* | *** Primary**  F:ATGGCGAGTGGTGGAATA  R:CCCAACTACGCTAAGGATT  ***Secondary**  F:TGTAAACGATGCCGACAGAG  R:CAACACTGAAGCCAATGCGAGC | 99 | KC594038.1 |
| Stray 23 | *P. hominis* | *** Primary**  F:ATGGCGAGTGGTGGAATA  R:CCCAACTACGCTAAGGATT  ***Secondary**  F:TGTAAACGATGCCGACAGAG  R:CAACACTGAAGCCAATGCGAGC | 99 | MH997492.1 |
| Stray 24 | *-* | - | - | Unidentified |
