# Supplementary material for: Metabarcoding study of potential pathogens and zoonotic risks associated with dog feces in Seoul, South Korea
Source: PLoS Negl Trop Dis. 2024 Aug 28;18(8):e0012441. doi: 10.1371/journal.pntd.0012441 (PMC11355564; doi:10.1371/journal.pntd.0012441)
Supplement: S7 Table — (DOCX) [file pntd.0012441.s011.docx]

Table S7. Comparison of the target gene sequence of *Giardia* between the Sanger sequencing result and the best match obtained from NCBI blastn analysis.

| Sample  No. | Identified Species (strains) | Primers | Percent Identity | Best Matches in GenBank |
| --- | --- | --- | --- | --- |
| Pet 6 | *Giardia intestinalis*  (assemblage D) | ***Primary**  F:AAGCCCGACGACCTCACCCGCAGTGC  R:GAGGCCGCCCTGGATCTTCGAGACGAC  ***Secondary**  F:GAACGAACGAGATCGAGGTCCG  R:CTCGACGAGCTTCGTGTT | 99 | KY979503.1 |
| Pet 15 | *G. intestinalis*  (assemblage D) | ***Primary**  F:AAGCCCGACGACCTCACCCGCAGTGC  R:GAGGCCGCCCTGGATCTTCGAGACGAC  ***Secondary**  F:GAACGAACGAGA  TCGAGGTCCG  R:CTCGACGAGCTTC  GTGTT | 100 | KY979497.1 |
|  | *G. intestinalis*  (assemblage C) | ***Primary**  F:AAATTATGCCTGCTCGTCG  R: CAAACCTTTTCCGCAAACC  ***Secondary**  F:CCCTTCATCGGTGGTAACTT  R:GTGGCCACCACTCCCGTGCC | 98 | KY979493.1 |
| Stray 4 | - | - | - | Unidentified |
| Stray 6 | - | - | - | Unidentified |
| Stray 7 | *G. intestinalis*  (assemblage C) | ***Primary**  F:AAGCCCGACGACCTCACCCGCAGTGC  R:GAGGCCGCCCTGGATCTTCGAGACGAC  ***Secondary**  F:GAACGAACGAGATCGAGGTCCG  R:CTCGACGAGCTTC  GTGTT | 99 | LC437428.1 |
| Stray 9 | *G. intestinalis*  (assemblage D) | ***Primary**  F:AAGCCCGACGACCTCACCCGCAGTGC  R:GAGGCCGCCCTGGATCTTCGAGACGAC  ***Secondary**  F:GAACGAACGAGA  TCGAGGTCCG  R:CTCGACGAGCTTC  GTGTT | 99 | KJ027418.1 |
|  | *G. intestinalis*  (assemblage C) | ***Primary**  F:AAATTATGCCTGCTCGTCG  R: CAAACCTTTTCCGCAAACC  ***Secondary**  F:CCCTTCATCGGTGGTAACTT  R:GTGGCCACCACTCCCGTGCC | 99 | MF974557.1 |
| Stray 10 | *G. intestinalis*  (assemblage D) | ***Primary**  F:AAGCCCGACGACCTCACCCGCAGTGC  R:GAGGCCGCCCTGGATCTTCGAGACGAC  ***Secondary**  F:GAACGAACGAGA  TCGAGGTCCG  R: CTCGACGAGCTTC  GTGTT | 99 | MK982549.1 |
|  | *G. intestinalis*  (assemblage C) | ***Primary**  F:AAATTATGCCTGCTCGTCG  R: CAAACCTTTTCCGCAAACC  ***Secondary**  F:CCCTTCATCGGTGGTAACTT  R:GTGGCCACCACTCCCGTGCC | 99 | AY228641.1 |
| Stray 11 | *G. intestinalis*  (assemblage C) | ***Primary**  F:AAGCCCGACGACCTCACCCGCAGTGC  R:GAGGCCGCCCTGGATCTTCGAGACGAC  ***Secondary**  F:GAACGAACGAGA  TCGAGGTCCG  R: CTCGACGAGCTTC  GTGTT | 99 | KX960130.1 |
| Stray 13 | *G. intestinalis*  (assemblage C) | ***Primary**  F:AAATTATGCCTGCTCGTCG  R: CAAACCTTTTCCGCAAACC  ***Secondary**  F:CCCTTCATCGGTGGTAACTT  R:GTGGCCACCACTCCCGTGCC | 100 | LC437516.1 |
| Stray 16 | *G. intestinalis*  (assemblage C) | ***Primary**  F:AAGCCCGACGACCTCACCCGCAGTGC  R:GAGGCCGCCCTGGATCTTCGAGACGAC  ***Secondary**  F:GAACGAACGAGA  TCGAGGTCCG  R: CTCGACGAGCTTC  GTGTT | 99 | KJ027413.1 |
| Stray 17 | *G. intestinalis*  (assemblage A) | ***Primary**  F:AAGCCCGACGACCTCACCCGCAGTGC  R:GAGGCCGCCCTGGATCTTCGAGACGAC  ***Secondary**  F:GAACGAACGAGA  TCGAGGTCCG  R: CTCGACGAGCTTC  GTGTT | 99 | KM926506.1 |
|  | *G. intestinalis*  (assemblage C) | ***Primary**  F:AAATTATGCCTGCTCGTCG  R: CAAACCTTTTCCGCAAACC  ***Secondary**  F:CCCTTCATCGGTGGTAACTT  R:GTGGCCACCACTCCCGTGCC | 99 | AY228641.1 |
| Stray 19 | *G. intestinalis*  (assemblage D) | ***Primary**  F:AAGCCCGACGACCTCACCCGCAGTGC  R:GAGGCCGCCCTGGATCTTCGAGACGAC  ***Secondary**  F:GAACGAACGAGA  TCGAGGTCCG  R: CTCGACGAGCTTC  GTGTT | 100 | MK982549.1 |
| Stray 20 | *G. intestinalis*  (assemblage D) | ***Primary**  F:AAGCCCGACGACCTCACCCGCAGTGC  R:GAGGCCGCCCTGGATCTTCGAGACGAC  ***Secondary**  F:GAACGAACGAGA  TCGAGGTCCG  R: CTCGACGAGCTTC  GTGTT | 99 | KJ027418.1 |
| Stray 21 | *G. intestinalis*  (assemblage D) | ***Primary**  F:AAGCCCGACGACCTCACCCGCAGTGC  R:GAGGCCGCCCTGGATCTTCGAGACGAC  ***Secondary**  F:GAACGAACGAGA  TCGAGGTCCG  R: CTCGACGAGCTTC  GTGTT | 99 | MK982549.1 |
| Stray 22 | *G. intestinalis*  (assemblage D) | ***Primary**  F:AAGCCCGACGACCTCACCCGCAGTGC  R:GAGGCCGCCCTGGATCTTCGAGACGAC  ***Secondary**  F:GAACGAACGAGA  TCGAGGTCCG  R: CTCGACGAGCTTC  GTGTT | 99 | MK982549.1 |
|  | *G. intestinalis*  (assemblage C) | ***Primary**  F:AAATTATGCCTGCTCGTCG  R: CAAACCTTTTCCGCAAACC  ***Secondary**  F:CCCTTCATCGGTGGTAACTT  R:GTGGCCACCACTCCCGTGCC | 99 | KX014797.1 |
| Stray 23 | *G. intestinalis*  (assemblage A) | ***Primary**  F:AAGCCCGACGACCTCACCCGCAGTGC  R:GAGGCCGCCCTGGATCTTCGAGACGAC  ***Secondary**  F:GAACGAACGAGA  TCGAGGTCCG  R: CTCGACGAGCTTC  GTGTT | 99 | KF963547.1 |
